# Subjective cognitive complaints at age 70: associations with amyloid and mental health

**DOI:** 10.1136/jnnp-2020-325620

**Published:** 2021-05-25

**Authors:** Ivanna M. Pavisic, Kirsty Lu, Sarah E. Keuss, Sarah-Naomi James, Christopher A. Lane, Thomas D. Parker, Ashvini Keshavan, Sarah M. Buchanan, Heidi Murray-Smith, David M. Cash, William Coath, Andrew Wong, Nick C. Fox, Sebastian J. Crutch, Marcus Richards, Jonathan M. Schott

**Affiliations:** 1 Dementia Research Centre, Queen Square Institute of Neurology, University College London, London, UK; 2 UK Dementia Research Institute, University College London, London, UK; 3 Medical Research Council Unit for Lifelong Health and Ageing, University College London, London, UK

## Abstract

**Objective:**

To investigate subjective cognitive decline (SCD) in relation to β-amyloid pathology and to test for associations with anxiety, depression, objective cognition and family history of dementia in the Insight 46 study.

**Methods:**

Cognitively unimpaired ~70-year-old participants, all born in the same week in 1946 (n=460, 49% female, 18% amyloid-positive), underwent assessments including the SCD-Questionnaire (MyCog). MyCog scores were evaluated with respect to ^18^F-Florbetapir-PET amyloid status (positive/negative). Associations with anxiety, depression, objective cognition (measured by the Preclinical Alzheimer Cognitive Composite, PACC) and family history of dementia were also investigated. The informant’s perspective on SCD was evaluated in relation to MyCog score.

**Results:**

Anxiety (mean (SD) trait anxiety score: 4.4 (3.9)) was associated with higher MyCog scores, especially in women. MyCog scores were higher in amyloid-positive compared with amyloid-negative individuals (adjusted means (95% CIs): 5.3 (4.4 to 6.1) vs 4.3 (3.9 to 4.7), p=0.044), after accounting for differences in anxiety. PACC (mean (SD) −0.05 (0.68)) and family history of dementia (prevalence: 23.9%) were not independently associated with MyCog scores. The informant’s perception of SCD was generally in accordance with that of the participant.

**Conclusions:**

This cross-sectional study demonstrates that symptoms of SCD are associated with both β-amyloid pathology, and more consistently, trait anxiety in a population-based cohort of older adults, at an age when those who are destined to develop dementia are still likely to be some years away from symptoms. This highlights the necessity of considering anxiety symptoms when assessing Alzheimer’s disease pathology and SCD.

## Introduction

Alzheimer’s disease (AD) has a preclinical window extending perhaps 20 years,^1^ characterised by accumulation of β-amyloid and tau pathology.^2^ Although individuals with preclinical AD are by definition cognitively unimpaired, they may experience subjective cognitive decline (SCD)—self-reported worsening of cognitive abilities despite performing normally on objective cognitive tests.^3^ Evidence suggests that SCD is associated with AD biomarkers, including β-amyloid pathology and structural and functional changes on MRI and PET,^4^ and with increased risk of cognitive decline,^3 5^ mild cognitive impairment (MCI) and dementia.^6^ SCD is often defined categorically (present/absent), but can also be considered on a continuous spectrum.^7^

Up to three-quarters of older adults with normal cognition self-report subjective decline,^8^ but the percentage of individuals with SCD meeting criteria for preclinical AD is rather variable.^9^ In disentangling this association, key factors are depression and anxiety, which are both associated with increased risk of incident MCI^10^ and with self-reported memory problems.^11^

This study aimed to investigate associations between SCD, symptoms of anxiety and depression, and amyloid status in a population-based sample at age ~70 years—when rates of dementia are low (~3%)^12^ but the prevalence of amyloid pathology is already significant (~15%–25%).^13^ We hypothesised that amyloid-positivity would be associated with greater SCD, after accounting for anxiety and depression. The proposed mechanism is that SCD reflects the cognitive consequences of accumulating pathology, but is independently associated with anxiety and depression (as individuals with anxiety and depression may be more likely to endorse SCD symptoms). We also investigated whether SCD symptoms were associated with lower objective neuropsychology scores and family history of dementia. Some studies have found higher SCD in individuals with a family history of AD, which may be due to inheritance of genetic risk factors and/or heightened vigilance to cognitive changes as a result of having witnessed cognitive decline in family members.^14 15^

## Methods

### Study design and participants

The Medical Research Council National Survey of Health and Development (NSHD) is a population-based cohort, originally comprised of 5362 individuals born across mainland Britain during 1 week in March 1946. With 24 data collections across their life course, it is the world’s longest continuously running birth cohort.^16^ For the Insight 46 neuroscience substudy, 502 NSHD participants were assessed at University College London between May 2015 and January 2018. Recruitment procedures and representativeness have been described previously.^17 18^ Measures included cognitive testing, clinical and physical examination, Aβ-PET imaging, brain MRI and other assessments detailed elsewhere.^18^

In line with Molinuevo *et al,* 19 recommendations for SCD research, participants with cognitive impairment (Mini Mental State Examination (MMSE^20^) <26) and major neurological/psychiatric conditions (which might result in subjective complaints due to an acute event) were excluded. Conditions included: epilepsy (requiring active treatment) (n=6); psychiatric disorders requiring antipsychotics or electroconvulsive therapy (n=4); traumatic brain injury or major neurosurgery (n=2); multiple sclerosis (n=3); stroke (n=18), radiologic evidence of possible brain malignancy (n=1) or any neurodegenerative disorder (n=8). Five participants had MMSE <26, all of whom already met criteria for a neurological disorder. In total 460 individuals were included in the study.

### SCD outcomes

Subjective cognition was measured using the MyCog questionnaire, a brief validated tool, part of the SCD-Questionnaire (SCD-Q).^21^ MyCog comprises 24 yes/no questions assessing perceived decline in instrumental activities of daily living over the preceding 2 years. We considered MyCog scores as a continuous spectrum of SCD symptoms; a higher score indicates greater perceived cognitive decline.^21^

As in the original SCD-Q, we preceded the MyCog with a series of questions about general perception of cognitive function, which were informed by the SCD-plus criteria^3^: (1) ‘Do you perceive memory or cognitive difficulties?’ (2) ‘In the last 2 years has your cognition or memory declined?’ If yes to (1) and/or (2): (3) ‘Do you perceive memory or cognitive difficulties more than other people the same age?’ (4) ‘At what age did these start?’ (5) ‘Would you ask a doctor about these difficulties?’ These questions were not designed for quantitative purposes but administered to provide an overview of the concerns and in order to establish whether participants wished for their general medical practitioner to be contacted based on these concerns.

To evaluate the informant’s perception of the participant’s cognition we used the AD8,^22^ a brief informant interview consisting of eight questions, sensitive to detecting early cognitive changes associated with dementia and which correlates with Clinical Dementia Rating (CDR) scores.^23^

### Biomarker measures

Aβ-PET and multimodal MRI data were collected simultaneously during a 60 min scanning session on a single Biograph mMR 3T PET/MRI scanner (Siemens Healthcare, Erlangen, Germany), with injection of 370 MBq of F^18^-Florbetapir (Amyvid).

Quantification methods have previously been described,^18 24^ but, in brief: Aβ deposition was quantified using the standard uptake value ratio (SUVR) calculated from cortical regions of interest with a reference region of eroded subcortical white matter. Aβ-PET attenuation correction was performed using a pseudo-CT method^18^; for 26 participants where this was not possible due to technical issues, we used a method based on the ultrashort echo time MRI sequence.^24^ A cut-point for amyloid-positivity was defined at SUVR >0.6104.^24^ Of the 460 participants included in this study, 40 were missing PET data (see^24^ for reasons).

*APOE-*ε4 genotype was determined from DNA analysis of blood samples.^18^ Individuals were classified as ε4-non-carrier (69.9%) or ε4-carrier (heterozygous: 27.5%; homozygous: 2.6%).

### Neuropsychological testing

The neuropsychology battery has previously been described,^24^ and was used to derive the Preclinical Alzheimer Cognitive Composite (PACC).^25^ We have previously shown a relationship between PACC score and life course variables,^24^ and so considered childhood cognitive ability, education and socioeconomic position (SEP) in our analysis.

### Life course and clinical variables

Childhood cognitive ability was measured at age 8 (or at ages 11 or 15 if earlier data were missing) using tests of verbal and nonverbal ability, standardised into a single *z*-score.^24^ These standardised scores were based on the full NSHD cohort.

Highest educational qualification achieved by age 26 was grouped into five categories: no qualification, below O-levels (vocational), O-levels and equivalents, A-levels and equivalents, higher education (degree/equivalents).^24^

SEP was derived from participants’ own occupation at age 53, or earlier if this was missing. Occupations were coded according to the UK Registrar General’s Standard Occupational Classification, in six categories: unskilled, partly skilled, skilled manual, skilled nonmanual, intermediate, professional.^24^

Two mental health measures were available: (1) the 28-item version of the General Health Questionnaire (GHQ)^26^ and (2) the State-Trait Anxiety Inventory (STAI).^27^ The GHQ was administered during the NSHD data collection at ages 68–69,^17^ measuring depression and general health with a validated threshold (≥5) indicating severity consistent with a ‘mental health disorder’ or caseness.^28^ The STAI contains 20 items that assess trait anxiety (how the individual feels generally) and 20 items that examine state anxiety (anxiety at the present moment), measured on the day as the MyCog. Each item is rated on a 4-point scale with higher scores indicate greater anxiety.

Participants were classified as having a family history of dementia if they reported one or more parent or sibling with a diagnosis of AD and/or ‘dementia not otherwise specified’. Given the particular focus on preclinical AD, diagnoses of other types of dementia (eg, vascular, frontotemporal, dementia with Lewy bodies) or other neurodegenerative or psychiatric conditions, were not included in the family history of dementia category. We asked participants to report age of onset of their relatives’ symptoms.

### Statistical analysis

Participant characteristics were initially compared between amyloid-positive and amyloid-negative groups using t-tests, or rank-sum tests where the distribution of the variable was skewed. Chi-squared tests were used to compare group proportions for any binary variables.

We evaluated relationships between the informant’s (AD8 score) and participant’s symptoms ratings using a multivariable regression model with MyCog as the outcome, AD8 score as the predictor and age and sex as covariates. In this analysis, all cognitively unimpaired participants (n=460) were included.

Similar to other reports,^29^ we used a multivariable linear regression model with SCD symptoms (MyCog) as the outcome and amyloid status and sex and age as predictors (model 1) to assess whether SCD symptoms were associated with amyloid status. We then added measures of anxiety and depression (models 2–4); the PACC measure of objective cognitive performance (model 5) and three life course variables—childhood cognitive ability, education and SEP—that have consistently shown direct and indirect associations with objective cognition throughout adulthood and are only moderately correlated with each other^24 30^ (model 6). Family history of dementia was considered as a final predictor (model 7).

Our multivariable linear regression models therefore contained the following predictors:

- Model 1=amyloid status, age, sex.
- Model 2=model 1+trait anxiety score.
- Model 3**=**model 2+state anxiety score.
- Model 4**=**model 3+GHQ (mental health disorder yes/no).
- Model 5**=**model 4+PACC.
- Model 6**=**model 5**+**childhood cognitive ability, education and SEP.
- Model 7=model 6+family history of dementia (yes/no).

These analyses were based on the 420 cognitively unimpaired participants with available PET data. Examination of residuals was performed to check model fits. For outcomes with skewed distributions, bootstrapping was used to produce bias-corrected and accelerated 95% CIs from 2000 replications.

Although findings on the relationship between sex and symptoms of SCD are equivocal,^31^ evidence of sex differences in anxiety,^32^ depression^33^ and AD prevalence^34^ exist. Therefore, we tested for sex differences in anxiety and depression (using Wilcoxon rank-sum and χ^2^ tests respectively) and for interactions between sex and anxiety and depression in our regression models. We also tested for sex differences in informant perspective using a χ^2^ test after dichotomising AD8 score based on a validated cut-off for informant concern (≥2 points).^22^ Finally, we investigated interactions between amyloid status and anxiety and depression, to see whether the effects of anxiety and depression on MyCog score differed between amyloid-positive and amyloid-negative groups.

All analyses were conducted using Stata V.14. Statistical significance was set at p<0.05.

## Results

### SCD symptoms

Participant characteristics are provided in table 1. Around half of participants reported that they perceived memory or cognitive difficulties or decline, but of these individuals, only 9% considered this to be worse than their peers, and only 2% reported that they would seek medical advice about these difficulties (table 1).

**Table 1:** Participant characteristics

	All participants	N=420	
		β-amyloid-positive	β-amyloid-negative
N	460	77	343
Sex, % female	49	44	51
Age at assessment, mean (SD) (range)	70.7 (0.70) (69.2 to 71.8)	70.6 (0.7) (69.4 to 71.8)	70.6 (0.7) (69.2 to 71.8)
MMSE, mean (SD) (range)*	29.3 (0.89) (26 to 30)	29.1 (1.0) (26 to 30)	29.3 (0.9) (26 to 30)
PACC, z-score, mean (SD) (range)*	−0.05 (0.68) (−2.43 to 1.72)	−0.10 (0.73) (−2.10 to 1.31)	0.08 (0.66) (-2.43 to 1.72)
Highest education qualification, %			
None	16.2	16.9	16.1
Below O-levels (vocational)	5.0	6.5	4.3
O-levels or equivalent	24.8	24.7	25.5
A-levels or equivalent	36.3	33.8	36.5
Degree or equivalent	17.8	18.2	17.9
Childhood cognitive ability mean (SD) (range)†	0.39 (0.74) (−1.60 to 2.50)	0.41 (0.74) (−1.59 to 2.47)	0.44 (0.74) (−1.37 to 2.50)
Adult socioeconomic position, %			
Unskilled	1.0	1.3	0.6
Partially unskilled	4.8	3.9	5.4
Skilled manual	9.6	9.1	9.3
Skilled non-manual	21.5	16.9	22.0
Intermediate	52.0	53.3	51.9
Professional	11.3	15.6	10.5
*APOE* status, % ε4-carrier‡§	30.1	59.7	23.2
SCD questions			
Perceive memory or cognitive difficulties (% yes)	44.8	49.4	45.2
Perceive decline in cognition or memory over past 2 years (% yes)	45.4	50.6	45.5
Difficulties or decline worse than peers (% yes)	8.8¶	12.8**	8.2††
Would seek medical advice (% yes)	2.3¶	6.4**	1.5††
SCD age onset, mean (SD) (range)	63.2 (10.3) (20 to 70) ¶	63.6 (10.2) (20 to 70)**	62.9 (10.7) (20 to 70)††
SCD ≥60 years, %	93.0	90.9	93.3
Total MyCog score (out of 24): mean (SD) (range)*	4.4 (3.9) (0 to 23)	5.2 (3.6) (0 to 15)	4.3 (3.9) (0 to 23)
AD8, mean (SD) (range)‡‡	0.2 (0.6) (0 to 5)	0.4 (1.0) (0 to 5)	0.1 (0.4) (0 to 3)
AD8, % AD8 ≥2*	4.1	10.4	2.6
Trait anxiety, mean (SD) (range)	31.8 (7.9) (20 to 65)	31.1 (8.0) (20 to 65)	31.9 (7.8) (20 to 64)
State anxiety, mean (SD) (range)	29.6 (7.9) (20 to 61)	29.3 (7.1) (20 to 52)	29.8 (8.1) (20 to 61)
Mental health disorder prevalence at age 69, % yes§§	7.0	10.4	6.2
Family history of dementia, % yes* ¶¶	23.9	33.8	22.5

*The difference between the amyloid groups for this variable was significant at p<0.05.

†Z-scores for childhood cognitive ability were based on the full National Survey of Health and Development cohort of n=5362, so the mean for Insight 46 participants indicates that they had higher childhood cognitive ability on average than their peers not recruited to this substudy.

‡The difference between the amyloid groups for this variable was significant at p<0.01.

§n=458 (the participants that had *APOE* status information available).

¶n=260 (the participants who answered ‘yes’ either to perceiving memory or cognitive difficulties or decline in cognition or memory in the last 2 years).

**n=47 (the participants who answered ‘yes’ either to perceiving memory or cognitive difficulties or decline in cognition or memory in the last 2 years).

††n=195 (the participants who answered ‘yes’ either to perceiving memory or cognitive difficulties or decline in memory or cognition in the last 2 years).

‡‡n=459 (one informant questionnaire was not completed).

§§n=452 (the participants that completed the GHQ-28 questionnaire).

¶¶Of these participants with a family history, their affected relatives were: mother=70.0%, mean age at onset (SD): 82.2 (9.1) years; father=28.2%, 78.5 (8.8) years; siblings=8.2%, 75.5 (10.0) years (percentages do not add up to 100% as some people had multiple relatives with a family history of dementia).

GHQ, General Health Questionnaire; MMSE, Mini Mental State Examination; PACC, Preclinical Alzheimer Cognitive Composite; SCD, subjective cognitive decline.

AD8 scores were significantly associated with MyCog scores, showing agreement between participant and informant perspectives: MyCog increased by 0.94 points (95% CI 0.25 to 1.63, p=0.007) for every 1-point increase in AD8. Informants of male participants were more likely to report concerns (χ^2^=6.35, p=0.012): of the 19 participants with AD8 ≥2, 15 were male.

### Associations with amyloid

**Figure 1:**
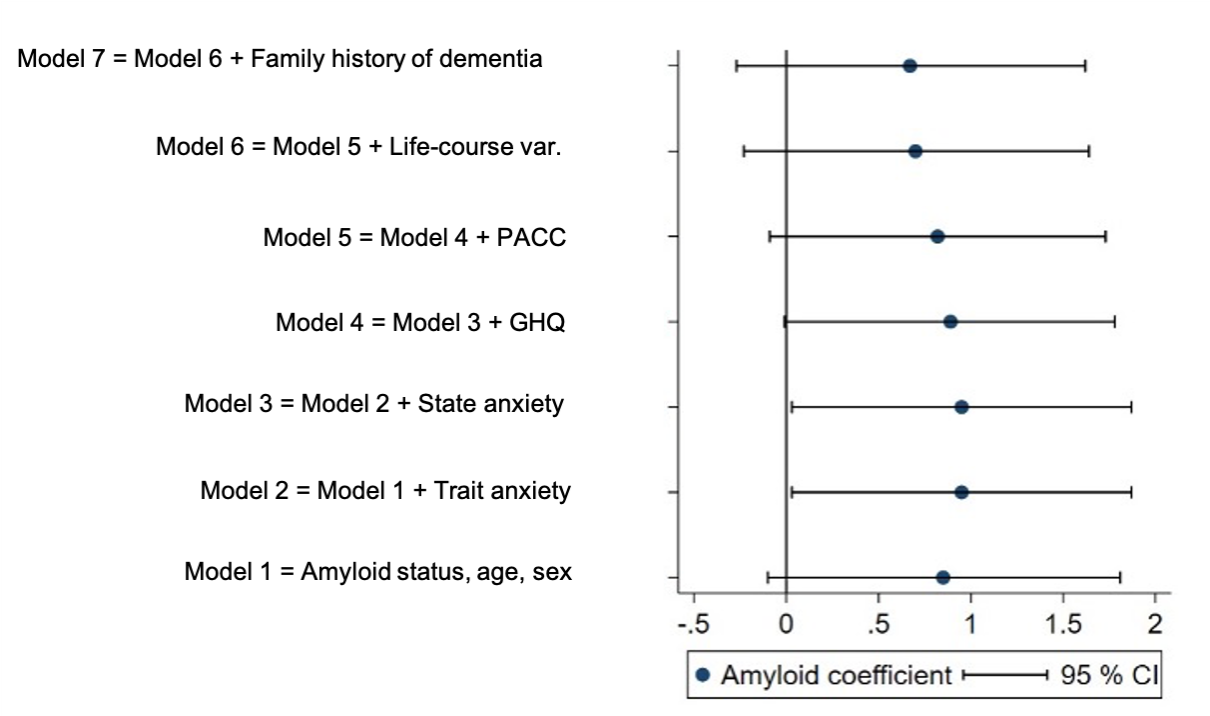
Amyloid coefficient as a predictor of MyCog score for each regression model. Amyloid coefficients (adjusted difference in mean MyCog score for amyloid-positive and negative groups) from each regression model with 95% CIs (n=420). Note that a positive coefficient indicates higher MyCog scores in the amyloid-positive group. GHQ, General Health Questionnaire; PACC, Preclinical Alzheimer Cognitive Composite.

**Figure 2:**
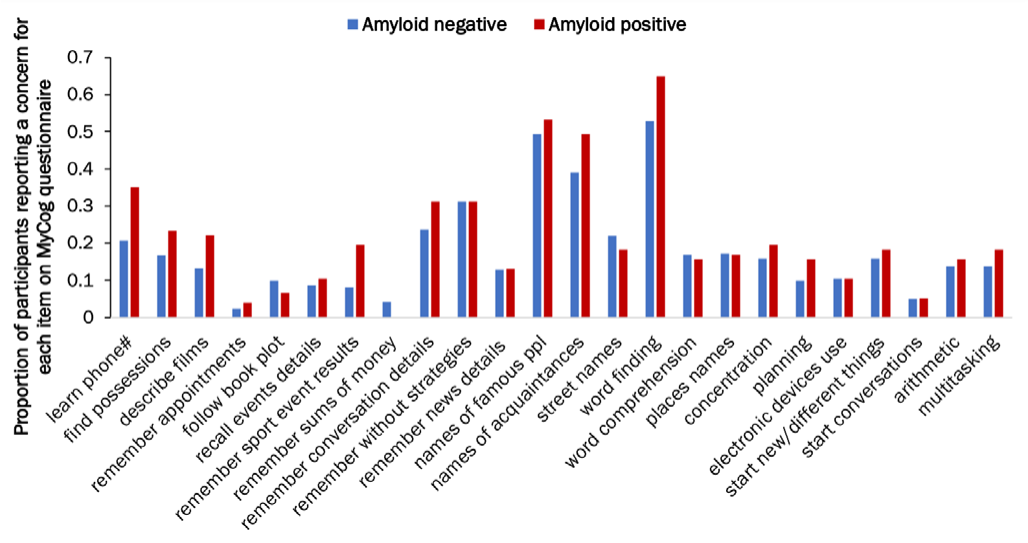
MyCog scores by β-amyloid status. Bar graphs shows the proportion of participants responding a concern for each MyCog item by β-amyloid status. Blue=Aβ- and Red=Aβ+.

In model 1, neither sex, age at assessment, nor amyloid status had a significant effect on MyCog scores (table 2). Although Aβ+individuals tended to have higher MyCog scores than Aβ-, this was not statistically significant (adjusted mean=5.2 (4.3 to 6.0) vs 4.3 (3.9 to 4.7), p=0.080, model 1, figure 1) (table 2, see also unadjusted means in table 1). The difference in MyCog scores between the amyloid groups was slightly greater in males than females, but this interaction was not statistically significant (interaction coefficient: −1.22 (-2.85 to 0.41), p=0.143). Aβ+participants reported greater concerns on most individual MyCog items compared with Aβ- (figure 2).

**Table 2:** Predictors of MyCog total score in n=420

	Coefficient and 95% CI for each model						
	Model 1	Model 2	Model 3	Model 4	Model 5	Model 6	Model 7
Amyloid status (negative as reference)	0.85 (-0.10 to 1.81)	0.95 (0.03 to 1.87) *	0.95 (0.03 to 1.87) *	0.89 (-0.01 to 1.78)	0.82 (-0.09 to 1.73)	0.70 (-0.23 to 1.64)	0.67 (-0.27 to 1.62)
Age	0.07 (-0.46 to 0.61)	0.05 (-0.48 to 0.57)	0.05 (-0.48 to 0.58)	0.05 (-0.49 to 0.60)	0.04 (-0.49 to 0.58)	0.19 (-0.37 to 0.75)	0.18 (-0.38 to 0.74)
Sex (male as reference)	0.19 (-0.54 to 0.92)	−0.10 (-0.80 to 0.60)	−0.10 (-0.80 to 0.60)	−0.14 (-0.85 to 0.56)	0.0005 (-0.71 to 0.71)	−0.03 (-0.84 to 0.77)	−0.03 (-0.83 to 0.78)
Trait anxiety		0.14 (0.08 to 0.19) †	0.14 (0.08 to 0.20) †	0.13 (0.07 to 0.19) †	0.13 (0.07 to 0.19) †	0.14 (0.08 to 0.21) †	0.14 (0.08 to 0.21) †
State anxiety			−0.002 (-0.05 to 0.05)	−0.003 (-0.06 to 0.05)	−0.007 (-0.06 to 0.05)	−0.02 (-0.07 to 0.04)	−0.02 (-0.07 to 0.04)
Mental health disorder (no as reference)				1.10 (-1.00 to 3.20)	1.05 (-1.02 to 3.11)	1.21 (-0.87 to 3.29)	1.20 (-0.90 to 3.29)
PACC *z*-score					−0.40 (-1.00 to 0.20)	−0.47 (-1.28 to 0.33)	−0.48 (-1.29 to 0.33)
Childhood cognitive ability						0.15 (-0.42 to 0.71)	0.14 (-0.43 to 0.71)
Education (per category)						0.12 (-0.25 to 0.48)	0.11 (-0.26 to 0.48)
SEP (per category)						0.02 (-0.41 to 0.45)	0.02 (-0.41 to 0.45)
Family history of dementia (no as reference)							0.25 (-0.67 to 1.17)
**R^2^**	0.008	0.084	0.084	0.088	0.093	0.102	0.103

Multivariable regression models were used so each association is independent of all others. R^2^ gives the proportion of variance in each cognitive outcome that is explained by the combined predictors.

*Significant at p<0.05;

†Significant at p<0.01.

PACC, Preclinical Alzheimer Cognitive Composite; SEP, socioeconomic position.

### Impact of anxiety and depression symptoms on SCD and amyloid associations

**Figure 3:**
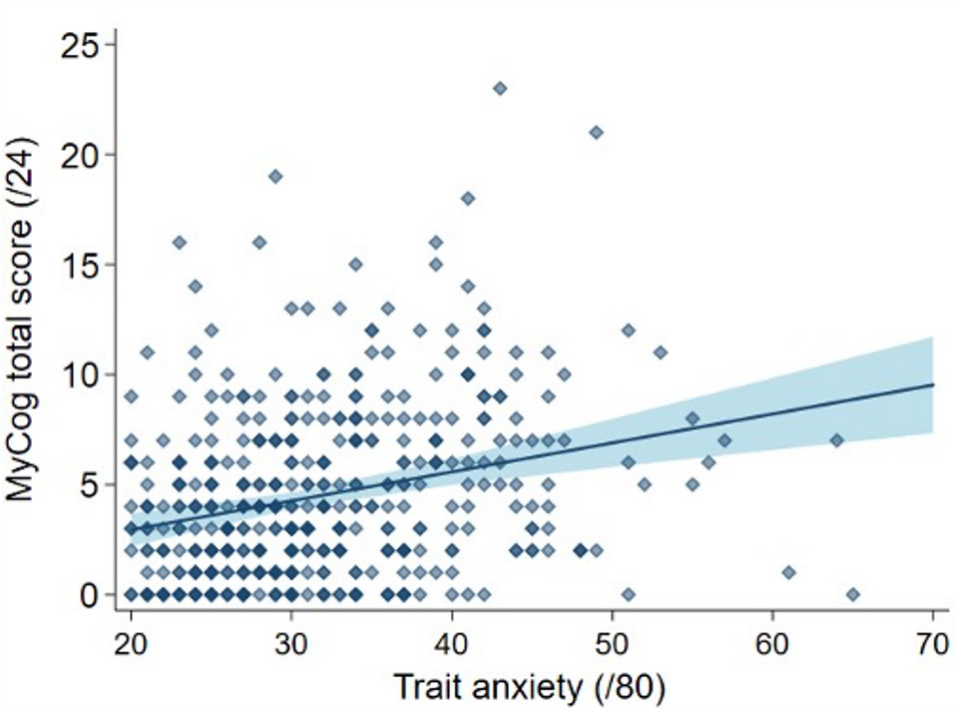
Total MyCog score against trait anxiety. Scatter plot shows the raw data (n=460) of MyCog against trait anxiety. The blue line is the line of best fit from the multivariable regression model (adjusted for sex, age at assessment and anxiety and depression symptoms, Model 4). The shaded areas represent 95% CIs. Note that the minimum possible trait anxiety score is 20.

There was no difference in anxiety or depression symptoms between amyloid groups (table 1). As expected, anxiety and depression measures showed positive associations with MyCog scores when examined separately and adjusted for age and sex (Trait anxiety: regression coefficient=0.14 (0.08 to 0.19) MyCog points per trait anxiety point, p<0.001; state anxiety: 0.07 (0.02 to 0.12), p=0.004; mental health disorder: 1.91 (−0.18 to 4.00) MyCog points higher for those who met criteria for mental health disorder compared with those who did not, p=0.074). However, trait anxiety was the only variable showing a significant association with MyCog when considering all mental health variables together (table 2, model 4, figure 1). This suggests the relationship between SCD symptoms and mental health variables was mostly explained by this factor (figure 3).

After adjustment for state and trait anxiety (models 2 and 3, figure 1), Aβ+ individuals had significantly higher MyCog scores compared with Aβ− (5.3 (4.4 to 6.1) vs 4.3 (3.9 to 4.7), p=0.044). This indicated that Aβ+ participants had greater concerns about their cognition above and beyond any differences in general anxious tendencies.

Further adjusting for GHQ scores slightly attenuated MyCog associations with amyloid (Aβ+=5.2 (4.4 to 6.0), Aβ-=4.4 (3.9 to 4.7), p=0.053, model 4, figure 1).

Females reported greater anxiety than males (mean trait anxiety score: males=30.6 (SD 7.5), range: 20–65, females=33.0 (8.2), 20–64, p=0.0007; mean state anxiety score: males=28.6 (7.5), 20–52, females=30.7 (8.1), 20–61, p=0.004) and higher prevalence of case-level depression symptoms (males 4.8%, females 10.9%, χ^2^=−5.88, p=0.015). An interaction test between sex and anxiety in model 4 revealed a steeper association between state anxiety and MyCog scores in females compared with males (interaction coefficient=0.09 (−0.01 to 0.19), p=0.078). A similar but non-significant interaction was observed for trait anxiety (0.09 (−0.03 to 0.20), p=0.134) and no interaction was observed for mental health disorder (−0.43 (−5.50 to 4.63), p=0.868). No significant interactions were observed between amyloid status and any of the mental health variables (model 4: trait anxiety: p=0.887; state anxiety: p=0.667; depression: p=0.368).

### Objective cognitive performance

As we have previously reported,^24^ individuals who were Aβ+had lower scores on the PACC compared with Aβ- (regression coefficient=−0.17 (−0.31 to −0.03), p=0.019). However, PACC scores were not independently associated with MyCog scores, although the coefficient was in the predicted direction (table 2). The inclusion of PACC in the model slightly attenuated the association between amyloid-positivity and higher MyCog scores (model 5, p=0.194, figure 1). Adjusting for life-course factors (childhood cognitive ability, education, and SEP) further attenuated the association between amyloid-positivity and higher MyCog scores (p=0.139), although the life course factors were not independently associated with MyCog scores when included together (model 6, figure 1), or when added to model 5 one at a time (childhood cognitive ability: 0.25 (−0.30 to 0.81], p=0.371), SEP: −0.11 (−0.52 to 0.29), p=0.588; education: 0.14 (−0.21 to 0.48), p=0.433).

### Family history of dementia

Aβ+individuals were more likely to have a family history of dementia (table 1). The affected relatives (mostly parents, but some siblings) had an average age of onset of 80.6 (SD 9.3) years. There was no association between total MyCog score and family history of dementia (p=0.595) (table 2, model 7, figure 1). The addition of family history to the model further attenuated the association between MyCog and amyloid status (table 2, p=0.162).

While the aim of this study was to assess influences on the MyCog score in a cognitively normal elderly cohort, a different but related question is to what extent MyCog might predict β-amyloid status at this stage. See online supplemental materials for this analysis: results were consistent with the linear regression approach, that is, MyCog score was a statistically significant predictor of amyloid-positivity once trait anxiety was accounted for (online supplemental table e1).

10.1136/jnnp-2020-325620.supp1 Supplementary data

## Discussion

In this large population-based sample of older adults of approximately the same age our key finding was that SCD symptoms, measured using MyCog, were significantly associated with amyloid-positivity, but only after accounting for symptoms of anxiety and before adjusting for objective cognition and life course factors (childhood cognitive ability, education and SEP). This is consistent with the hypothesis that SCD symptoms are an early sign of AD, measurable at preclinical stages and correlating with accumulating pathology.^35^ Our results suggest that subtle manifestations of subjective cognitive concerns are detectable at age 70, more than a decade away from the estimated median age of onset of dementia in the UK.^36^ However, the increase of less than one point in MyCog scores from Aβ- to Aβ+ is small, reflecting the generally low MyCog scores in this cohort. In this context, it is perhaps relevant that around half of this cohort reported subjective cognitive difficulties (lower percentage compared with cohorts of similar age^8^) but very few of these individuals said that this was worse than their peers or that they would report their concerns to a doctor. Long-term prospective studies in individuals who eventually developed dementia suggest that SCD occurs on average ~10 years before dementia diagnosis.^37^ As the mean age of onset of SCD symptoms in our sample was 63.2 (10.3) years, longitudinal follow-ups might further unveil the specificity and sensitivity of SCD symptoms as markers for preclinical AD.

While all mental health measures were associated with MyCog scores in isolation, only trait anxiety showed an independent association. Consistent with a recent study that found no association between anxiety and amyloid or tau pathology (but did observe an association between repetitive negative thinking and greater pathological burden^38^), anxiety levels did not differ between the amyloid groups. This highlights the complexities of interpreting SCD symptoms in a clinical context, as individuals seeking medical help for their cognitive concerns may have diverse profiles of anxiety and depression symptoms, and current evidence does not support predictions about risk of progression from SCD to dementia on an individual basis.^39^ Regarding why adjusting for trait anxiety appeared to unmask an association between SCD and amyloid, we hypothesise that the MyCog questions captured general aspects of anxiety traits, particularly around low estimation of one’s own abilities, and therefore, accounting for these tendencies increased the sensitivity of the model to detect concerns specific to cognitive decline. However, as this relationship was no longer statistically significant when other predictors were taken into account, and given the limitations of our approach (see below), further studies are needed to disentangle the complex interactions between these variables.

In agreement with the literature on mental health and sex differences,^32 33^ females reported higher anxiety scores and a higher prevalence of case-level symptoms than males. As women are at a greater risk of developing AD, and mental health problems are associated with a greater risk of dementia, it is possible that anxiety differences may play a role in the disparity in dementia risk between sexes.^34^ We found female participants to have better cognition from the perspective of their informants (lower AD8 scores), consistent with our finding that females performed 0.39 SD higher than males on the PACC.^24^ However, we did not find an overall sex difference in participant-reported SCD symptoms.

While amyloid-positivity was associated with symptoms of SCD in our study and has previously been related to poorer objective cognitive performance in this cohort,^24^ there was no statistically significant relationship between MyCog score and objective cognitive performance after accounting for amyloid status. Comparing subjective and objective cognitive performance is inherently complex. As argued by Jessen,^40^ SCD symptoms may be independent of objective cognitive performance because the latter represents a cross-sectional measure whereas the former describes change over a time period (often years). For example, an individual whose cognition has declined from a high baseline level may perform equally to an individual whose cognition has remained stable at a lower level. Longitudinal follow-up of Insight 46 participants will address the question of whether self-perceived decline is related to change in performance on objective cognitive tests.

Our results show a similar prevalence (~25%) of family history of dementia to other studies of participants of a similar age^14^ but no association between family history and symptoms of SCD.

This study has a number of limitations. First, NSHD participants are all white,^24^ limiting the generalisability of our findings to more diverse populations. Second, participants with missing neuroimaging data were more likely to have mental health problems^17^ and we excluded individuals with major neurological and psychiatric conditions, so individuals with mental health problems are under-represented in our analyses. Third, pathologies other that β-amyloid including vascular disease or alpha-synuclein may also be determinant of SCD. Lastly, limitations associated with the approach chosen include: loss of information and increased risk of false positives when dichotomising variables (eg, amyloid status or mental health disorder) as well as the greater risk of multiplicity problems with a stepwise regression approach. Strengths of this study include the large sample size and the very small age-range meaning that our findings are robust to the confounding effects of age-related changes in SCD.

## Conclusions

In summary, our findings show independent effects of trait anxiety and β-amyloid status on symptoms of SCD in cognitively normal ~70 years old. This suggests that the presence of subjective cognitive symptoms may have some utility in identifying people at risk of developing AD dementia in older age, provided the influence of anxiety symptoms is carefully considered.

## Data Availability

Data are available on reasonable request. Anonymised data will be shared by request from qualified investigators (skylark.ucl.ac.uk/NSHD/doku.php).
