# Supplementary material for: Subjective cognitive complaints at age 70: associations with amyloid and mental health
Source: J Neurol Neurosurg Psychiatry. 2021 May 25;92(11):1215–21. doi: 10.1136/jnnp-2020-325620 (PMC8522456; doi:10.1136/jnnp-2020-325620)
Supplement: Supplementary data [file jnnp-2020-325620supp001.pdf]

Subjective cognitive complaints at age 70: Associations with amyloid and mental health – Pavisic et al., 2021

Supplementary Materials

While the aim of this study was to assess influences on the MyCog score in a cognitively unimpaired elderly cohort, a different but related question is: to what extent MyCog might predict β-amyloid status at this stage? Understanding whether SCD symptoms may predict amyloid status has clinical relevance as it would be very useful in reducing the need for expensive or invasive methods such as amyloid PET.

To investigate this, we used logistic regression with β-amyloid as a dichotomous outcome. The models were as follows:

- **Model 1** = MyCog, age, sex
- **Model 2** = Model 1 + trait anxiety score
- **Model 3 =** Model 2 + state anxiety score
- **Model 4 =** Model 3 + GHQ (mental health disorder yes/no)
- **Model 5 =** Model 4 + PACC

Results are presented in Table e1. Overall, we found results consistent with the linear regression approach, i.e. that including age and sex as covariates MyCog score alone was not a statistically significant predictor of being amyloid-positive, but that this was predictive once trait anxiety was accounted for and before adjusting for objective cognition.

Pavisic IM*, et al. J Neurol Neurosurg Psychiatry* 2021; 92:1215–1221. doi: 10.1136/jnnp-2020-325620

Subjective cognitive complaints at age 70: Associations with amyloid and mental health – Pavisic et al., 2021

Table e1. Predictors of amyloid status in n=420

	**Odds Ratio and 95% confidence interval for each model (Amyloid negative as reference)**				
	**Model 1**	**Model 2**	**Model 3**	**Model 4**	**Model 5**
MyCog score	1.05 [0.99, 1.12]	1.07 [1.00, 1.14] *	1.07 [1.00, 1.14] *	1.06 [1.00, 1.13]	1.05 [0.99, 1.12]
Age	1.01 [0.70, 1.43]	1.00 [0.70, 1.44]	1.00 [0.70, 1.44]	1.03 [0.71, 1.48]	1.01 [0.70, 1.47]
Sex (male as reference)	0.76 [0.46, 1.25]	0.79 [0.48, 1.33]	0.80 [0.48, 1.32]	0.79 [0.47, 1.32]	0.89 [0.53, 0.51]
Trait anxiety		0.98 [0.94, 1.01]	0.98 [0.94, 1.02]	0.98 [0.94, 1.02]	0.98 [0.94, 1.02]
State anxiety			1.00 [0.96, 1.04]	1.00 [0.96, 1.04]	1.00 [0.96, 1.04]
Mental health disorder (no as reference)				1.86 [0.76, 4.59]	1.79 [0.72, 4.45]
PACC *z*-score					0.70 [0.48, 1.03]
**Pseudo R^2**	0.010	0.014	0.014	0.018	0.026

Logistic regression models were used so each association is independent of all others. The pseudo R^2 gives the proportion of variance in each cognitive outcome that is explained by the combined predictors. SEP = socioeconomic position; PACC=Preclinical Alzheimer's composite score. * Significant at $p < 0.05$; **Significant at $p < 0.01$

Pavisic IM, *et al. J Neurol Neurosurg Psychiatry* 2021; 92:1215–1221. doi: 10.1136/jnnp-2020-325620
